# Draft Genome Sequence of *Thermanaeromonas* sp. Strain C210, Isolated in the Presence of Carbon Monoxide

**DOI:** 10.1128/MRA.00608-20

**Published:** 2020-08-13

**Authors:** Masao Inoue, Ayumi Tanimura, Yuto Fukuyama, Suguru Okunishi, Hiroto Maeda, Takashi Yoshida, Yoshihiko Sako

**Affiliations:** a Graduate School of Agriculture, Kyoto University, Sakyo-ku, Kyoto, Japan; b Faculty of Fisheries, Kagoshima University, Kagoshima, Japan; Georgia Institute of Technology

## Abstract

The genus *Thermanaeromonas* comprises two species of thermophilic, strictly anaerobic, spore-forming bacteria. Here, we report the draft genome sequence of *Thermanaeromonas* sp. strain C210, which was first isolated in the presence of carbon monoxide. The genome sequence provides insight into carbon monoxide-dependent metabolism for members of the genus *Thermanaeromonas*.

## ANNOUNCEMENT

The genus *Thermanaeromonas* is a member of the family *Thermoanaerobacteraceae*, class *Clostridia*, phylum *Firmicutes*. In this genus, two species, T. toyohensis and T. burensis, are described as thermophilic, strictly anaerobic, spore-forming, thiosulfate-reducing bacteria (1, 2); however, genomic information is available only for *T. toyohensis* strain ToBE^T^ (GenBank accession number LT838272). Here, we report the draft genome sequence of *Thermanaeromonas* sp. strain C210, which was first isolated from marine sediment in the presence of carbon monoxide (CO).

The sediment was collected from Yamagawa Bay, Ata Caldera, in Kagoshima Prefecture, Japan (31°12′43″N, 130°38′18″E; depth, 10 to 11 cm; elevation, −46 m). Strain C210 was enriched, isolated, and cultivated for genome sequencing at 65°C under 100 % CO gas in hypotonic artificial seawater (hASW) medium, as described previously (3).

Genomic DNA extraction, library preparation, sequencing, and genome assembly were carried out as described previously (4, 5). Genomic DNA was extracted using the DNeasy blood and tissue kit (Qiagen, Hilden, Germany); then, a DNA library was prepared using the Nextera mate pair library preparation kit (Illumina, San Diego, CA). Sequencing was performed on the Illumina MiSeq instrument with the MiSeq reagent kit v.3 (600 cycles), which generated 4,827,688 paired-end reads. Quality trimming and adapter removal were performed using Trimmomatic v.0.3.6 (ILLUMINACLIP:2:30:10 LEADING:3 TRAILING:3 SLIDINGWINDOW:4:15 MINLEN:30) (6). Mate pair reads were selected and junction adapters were trimmed using NxTrim v.0.4.1 (7). *De novo* genome assembly was performed with SPAdes v.3.13.0 with the option “--hqmp” (8), using the filtered 2,314,613 mate pair reads. The assembled scaffolds were quality controlled using BWA v.0.7.17 (9), SAMtools v.0.1.19 (10), and NxRepair v.0.13 (11). Annotation was performed with the DFAST server v.1.0.2 (12). Genomic comparison was performed using OAT v.0.93.1 (13), GGDC v.2.1 (14), and BLAST (15). Default parameters were used for all software unless otherwise noted.

The draft genome was assembled into 19 scaffolds with an *N*_50_ value of 658,646 bp, an average coverage of 278×, a total length of 2,676,999 bp, and an average G+C content of 57.1%. The numbers of predicted protein-coding genes, rRNAs, and tRNAs were 2,618, 6, and 48, respectively. The average nucleotide identity and digital DNA-DNA hybridization to *T. toyohensis* ToBE^T^ were 71.4% and 24.0%, respectively. The sequence identities of the 16S rRNA genes to *T. toyohensis* ToBE^T^ and *T. burensis* IA106^T^ were 92.7% and 98.7 to 98.8%, respectively. These results suggest that strain C210 is a member of the genus *Thermanaeromonas*.

We identified three genes encoding anaerobic CO dehydrogenase (CODH), one with a gene cluster for hydrogen-evolving hydrogenase (TAMC210_016640 to TAMC210_16840), another with a gene cluster for acetyl-coenzyme A (CoA) synthase (TAMC210_19150 to TAMC210_19280), and the last with genes for CooF and ferredoxin-NAD(P)H oxidoreductase (TAMC210_04640 to TAMC210_04620). The amino acid sequence identities of these three CODHs of strain C210 to their orthologs in *T. toyohensis* ToBE^T^ were >85%. These results were consistent with the fact that strain C210 was enriched in the presence of CO and with recent bioinformatics-based analyses showing that the genus *Thermanaeromonas* includes possible hydrogen-evolving, CO-oxidizing bacteria (16–18).

### Data availability.

This draft genome sequence was deposited in GenBank under accession number BLWF00000000.1. The raw reads were deposited in SRA under accession number DRA010181.
